# Atypical Presentation of Disseminated Zoster in a Patient with Rheumatoid Arthritis

**DOI:** 10.1155/2015/124840

**Published:** 2015-10-05

**Authors:** Nirav Patel, Davinder Singh, Krunal Patel, Shadab Ahmed, Prachi Anand

**Affiliations:** Department of Medicine, Nassau University Medical Center, East Meadow, NY 11554, USA

## Abstract

Patients with rheumatoid arthritis (RA) have 2-fold increased risk of herpes zoster. In literature, limited information exists about disseminated cutaneous zoster in RA patients. An 83-year-old African-American female with RA presented with generalized and widespread vesicular rash covering her entire body. Comorbidities include hypertension, type II diabetes, and dyslipidemia. Patient was on methotrexate 12.5 mg and was not receiving any corticosteroids, anti-TNF therapy, or other biological agents. The patient was afebrile (98 F) with no SIRS criteria. Multiple vesicular lesions were present covering patient's entire body including face. Lesions were in different stages, some umbilicated with diameter of 2–7 cm. Many lesions have a rim of erythema with no discharge. On admission, patient was also pancytopenic with leukocyte count of 1.70 k/mm^3^. Biopsies of lesions were performed, which were positive for Varicella antigen. Subsequently, patient was started on Acyclovir. The patient's clinical status improved and rash resolved. Our patient presented with “atypical” clinical picture of disseminated cutaneous zoster with no obvious dermatome involvement. Disseminated zoster is a potentially serious infection that can have an atypical presentation in patients with immunocompromised status. High index of suspicion is needed to make the diagnosis promptly and to initiate therapy to decrease mortality and morbidity.

## 1. Introduction

Herpes zoster called shingles is the result of reactivation of latent varicella-zoster virus [1]. More than 90% of cases of herpes zoster occur in immunocompetent patients; however, risk increases by 20 to 100 times in immunocompromised patients. Immunosuppressive conditions associated with increased rates include HIV infection, organ transplant recipients, and malignancy (especially lymphoproliferative disorder) [2].

Rheumatoid arthritis patients have a 2-fold increased risk of herpes zoster compared to general population [3]. RA disease severity, disease modifying antirheumatic drugs (DMARDs), and biological agents have been associated with herpes zoster [4, 5]. Immunocompromised patients are at increased risk of developing complication of herpes zoster infection including dissemination and visceral organ involvement [6]. In our literature search, there was limited information about disseminated cutaneous zoster in RA patient. In this report, we would like to present a case of an atypical presentation of cutaneous disseminated zoster in a patient with RA on Methotrexate.

## 2. Case Report

### 2.1. History

An 83-year-old African-American female with rheumatoid arthritis presented with generalized and widespread vesicular rash covering her entire body. Comorbidities include hypertension, type II diabetes, and dyslipidemia. Patient had no fevers or chills; review of systems was otherwise negative. The patient was diagnosed with RA and is on low-dose Methotrexate 12.5 mg weekly for the past 8 years. Patient did not receive any corticosteroids, anti-TNF, or other biological therapy in last few years.

### 2.2. Physical Examination

The patient was afebrile (98 F) and did not meet Systemic Inflammatory Response Syndrome criteria [7]. Multiple vesicular lesions were present covering patient's entire body including face. Lesions were dark colored, in different stages, some umbilicated with diameter of 2–7 cm. Many lesions have a rim of erythema with no discharge. Patient did not have enlarged liver or any central nervous system involvement.

### 2.3. Hospital Course

On admission, patient was also pancytopenic with leukocyte count of 1.70 k/mm^3^. Patient had positive urine culture for* Klebsiella pneumoniae* and blood cultures were negative. Patient received 3 days of Ceftriaxone for uncomplicated urinary tract infection. Biopsies of lesions were performed on day 3 of admission by dermatology, which were positive for varicella antigen. Subsequently, patient was started on Acyclovir IV 700 mg BID for 3 days and later switched to PO 800 mg BID for 4 more days. Over the next 3 days, the patient's clinical status improved and rash improved. Patient did not have any relapses of herpes zoster infection or any sequela from infection.

## 3. Discussion

There is an apparent increase in the incidence of herpes zoster in patient with RA relative to general population [3]. There is no significant difference in severity of herpes zoster in patient RA compared to general population [3]. Dissemination of herpes zoster is defined as more than 20 vesicles outside primary and adjacent dermatomes [8]. Our patient presented with “atypical” clinical picture of disseminated cutaneous zoster with no obvious primary dermatome involvement.

In our patient, advance age, diabetes mellitus, rheumatoid arthritis, and Methotrexate could have contributed to dissemination of herpes zoster. Methotrexate have been implicated as a risk factor for developing infection and varicella-zoster infection [9, 10]. However, the effect of withholding or continuation of Methotrexate has not been studied. Therefore, in our patient, low-dose Methotrexate was continued.

Patients with dissemination of herpes zoster are at increased risk for end organ involvement, particularly lungs, liver, and brain [6]. Other complications include cornel ulceration, bacterial superinfection, and postherpetic neuralgia [6]. Therefore, identification and early treatment are important to decrease morbidity and mortality.

Treatment of choice for disseminated zoster is IV Acyclovir 10 mg/kg every 8 hours for 5–7 days. Infectious agents like bacteria and viral infection can induce pancytopenia [11, 12]. Certain virus can cause direct damage to stem cells and cause aplasia and the best documented one is parvovirus B19 [11, 12]. Our patient also presented with pancytopenia that improved upon initiation with Acyclovir.

## 4. Conclusion

Disseminated zoster is potentially serious infection that can have an atypical presentation. High index of suspicion is needed to make the diagnosis promptly and to initiate IV Acyclovir to decrease mortality and morbidity.
